# The Effect of the COVID-19 Pandemic on Emergency Department Visits for Neurological Diseases in Saudi Arabia

**DOI:** 10.7759/cureus.12200

**Published:** 2020-12-21

**Authors:** Ahmed K Bamaga, Omar Alharbi, Majed Bajuaifer, Abdulrahman Batarfi, Khalid H Althobaiti, Bader AlQusaibi

**Affiliations:** 1 Neurology: Pediatrics, King Abdulaziz University Hospital, King Abdulaziz University, Jeddah, SAU; 2 Medicine, King Abdulaziz University, Jeddah, SAU; 3 Neurology, King Abdulaziz University, Jeddah, SAU

**Keywords:** stroke, epilepsy, weakness. emergency visits, weakness

## Abstract

Introduction

COVID-19 has been a gravitating topic in the past months, yet much information about this new virus is to be unraveled. The uncertainties about the virus and its effects have affected a lot of daily life activities. One of these affected activities is emergency department (ED) visits and how this disease might have changed people's perspective on when to go to an emergency. This study aims to assess the effect of the COVID-19 pandemic on emergency department visits for neurological conditions.

Methods

A retrospective record review study was conducted at King Abdul-Aziz University Hospital (KAUH) during the month of July 2020. The study included visits of patients with common neurological conditions (headache, seizures, and weakness), during December 2019 - May 2020 at KAUH. Information obtained from the medical records included demographic data, date of visit, the reason for the visit, history of a similar episode, number of ED visits during the past year, priority given at the ED, length of hospitalization, diagnosis of COVID-19 at KAUH, known chronic diseases, and whether brain imaging was performed with which kind of imaging. Descriptive analysis was conducted to assess the impact of the pandemic on ED visits and statistical analysis (chi-square test) was performed on ED visit data to assess for significance.

Results

There was a 24% reduction in the number of visits for common neurological symptoms (during the pandemic) time period in comparison to (pre-pandemic). However, some other variables have also shown an increase (during the pandemic) time period. Most notably, brain CT scans, which underwent an 11.3% increase during the pandemic time period (p=0.005). Some variables have shown no significant change, for example, the relationship between the time period and the reason for the visit (p=0.305).

Conclusion

Multiple factors most likely contributed to the decrease in emergency department visits recorded in this study. One of the main reasons is the fear of catching COVID-19 infection by just vising the hospitals. Considering these findings, it is predominant to raise awareness when patients do need to go to the emergency department due to an acute neurological condition regardless of any pandemic.

## Introduction

Neurological problems are among the common medical emergencies presenting to the emergency departments (ED). Stroke, seizures, headache, and acute weakness are the more frequently encountered complaints. As one study reports, around 5% of all emergency department visits are due to neurological symptoms [1].

Weakness is a common presentation for a stroke or cerebrovascular accident (CVA). Triggered by ischemia (severely reduced blood flow) or blockage (thrombosis, arterial embolism) or a hemorrhage (brain blood vessel leaks or ruptures) [2]. Old age, high blood pressure, prior stroke, or transient ischemic attack (TIA), high cholesterol, diabetes, tobacco smoking, and atrial fibrillation has been reported in a study to be major risk factors [3-5]. Stroke has been one of the most significant causes of severe, long-term neurological impairment, and physical disability and is a common cause of death globally. Fifteen million people worldwide suffer from strokes every year, according to the World Health Organization (WHO). Of these, 5 million die and 5 million more are permanently disabled [6]. In Saudi Arabia, stroke is seen as a rapidly growing problem and a significant cause of illness and death. It is one of Saudi Arabia's most crucial social, economic, and medical problems [7]. One study reports the crude incidence of stroke to be 29.8/100,000/year in Saudi Arabia [8].

A seizure is a transient occurrence of signs and/or symptoms due to abnormal excessive or synchronous activity in the brain. It may be either acute symptomatic or unprovoked [9]. Generalized seizures are divided into subtypes, which are absence, generalized tonic colonic (GTC), myoclonic, and atonic [10]. According to the WHO 2010 Global Burden of Disease Study, it is the second most neurological disorder around the world in terms of disability-adjusted life years [11]. Seizures are a common neurological emergency that affects 9%-10% of the population [12]. In fact, one study showed that the incidence of acute symptomatic seizures was 29-39 per 100,000 per year [13]. While another study reported that the annual cumulative incidence of epilepsy was 67.77 per 100,000 persons (95% CI 56.69-81.03) while the incidence rate was 61.44 per 100,000 person-years (95% CI 50.75-74.38) [14]. While not many regional studies have reported the incidence of seizures, one study reported that the prevalence of epilepsy in Saudi Arabia was 6.5/1,000 [15].

Headache is the most common neurological condition, and it’s among the top 10 causes of disability [16], as over 45 million suffer from recurrent headaches in the U.S. alone [17]. The prevalence in Saudi Arabia is reported to be around 8%-12% [18].

In the second week of December 2019, coronavirus 2 (severe acute respiratory syndrome coronavirus 2 or SARS-CoV-2) first emerged in a small local fish and wild animal market in Wuhan city, China. The WHO stated that the symptoms and disease manifestations of SARS-CoV-2 were to be named as coronavirus 19 (COVID-19). COVID-19 symptoms may range from normal cold and influenza to a severe critical form, depending on the persons’ state of health. Individuals who suffer from diabetes, heart, and lung disease are likely to develop the critical forms of COVID-19, which lead to death. In March 2020, the WHO has declared the coronavirus outbreak as a global pandemic. The drastic spread of COVID-19 has been a matter of concern, infecting 1,948,617 patients in 210 countries and territories around the world, and causing around 121,846 deaths worldwide on April 14, 2020 [19-20].

During this pandemic, many countries are in lockdown and some governments have ordered curfews, all in aim to contain the COVID-19 outbreak. With that being said, some studies have shed light on different aspects of how this pandemic is effecting our health care systems, for example, one study conducted in a trauma center in New Zealand compared the rate of admissions two weeks before and two weeks after the New Zealand government declared national lockdown. They reported a 48% reduction in all injury-related admissions to the trauma center [21]. Emergency departments around the world have been heavily impacted in several ways; an example of such impact is hospitals in Daegu, Korea, where ER departments in some hospitals have been temporarily closed to limit nosocomial transmission [22-23].

Few studies have shed light on how the presentation and visit volume of these neurological diseases to the ED could be affected during this pandemic. One study done at London’s regional stroke center, serving a population of 1.8 million in Ontario, Canada, aimed to assess the impact of the COVID-19 pandemic on code stroke activation in the ED (May 26, 2020). They found a 20% drop in the number of code strokes in 2020 as compared to 2019, immediately after the first cases of COVID-19 were officially confirmed [24]. Another study conducted in London shows that attendance at accident and emergency departments in the UK has been widely reported to have decreased precipitously since the national lockdown was introduced on March 23, 2020 [25].

On the other hand, COVID-19 is a new disease. It’s relation to neurological diseases has not been thoroughly investigated yet, but the development of brain inflammation, confusion, seizures, and strokes have all been reported in some COVID-19 patients [26]. With that being said, some studies actually suggest a higher rate of cerebrovascular complications in patients with COVID-19 infection [27]. The impacts of this pandemic together with all its aspects and how they affect ED visits for the aforementioned neurological diseases have not been thoroughly studied yet.

This study aims to assess the effect of the COVID-19 pandemic on ED visits related to stroke, seizures, and weakness by comparing pre-COVID-19 (December 2019 - February 2020) to COVID-19 months (March-May 2020).

## Materials and methods

Study design and participants

This retrospective record review study was conducted during the month of July 2020 at King Abdul-Aziz University Hospital (KAUH) in the department of pediatrics and approved by the research ethics committee of KAUH (Reference No 384-20). This study included all patients evaluated for common neurological symptoms (headache, seizures, and weakness) during the time frame of December 2019 - May 2020 at the emergency department at KAUH. 1773 hospital records were reviewed of whom 494 visits were during December 2019 - May 2020.

Data collection

Information obtained from the medical records included demographic data as well as the date of visit, the reason for the visit, history of a similar previous episode, number of emergency department visits during the past year, priority given at the emergency department, length of hospitalization, diagnosis of COVID-19 at KAUH, known chronic diseases, and whether brain imaging was performed with the type of imaging and the finding. The description of the number of visits with neurological complaints in regards to the date of the visit was considered the primary outcome of this study.

The Statistical Package for the Social Sciences (SPSS) version 21 (IBM Corp., Armonk, NY) was used for statistical analysis. Mean and standard deviation was calculated to describe continuous variables while numbers and percentages were used for categorical variables. Descriptive analysis and chi-square tests were used to evaluate the data and find associations between different categorical variables. A p-value of less than 0.05 was considered significant.

## Results

Results from data analyzed to assess the effect of the COVID-19 pandemic on emergency department visits for common neurological symptoms showed that the total number of visits was 493, of which 281 were in the (pre-pandemic) time period while 212 visits were in the during-pandemic time period. Demographic and other neurological and emergency department variables are reported in Table 1. All variables were compared regarding the two different time periods (Table 2).

**Table 1:** Patients demographics

	Number	Percentage	Valid percentage
Gender:			
Male	235	47.70%	47.70%
Female	258	52.30%	52.30%
Nationality:			
Saudi	285	57.80%	57.80%
Non-Saudi	208	42.20%	42.20%
Age group:			
0-14 years	66	13.40%	13.40%
15-64 years	331	67.10%	67.10%
≥ 65 years	96	19.50%	19.50%
Reason for visit:			
Headache	220	44.60%	45.60%
Seizures	118	23.90%	24.50%
Weakness	86	17.40%	17.80%
Dizziness	21	4.30%	4.40%
Decreased LOC	24	4.90%	5%
Other neurological complaints	13	2.60%	2.70%
Visit during government lockdown:			
Yes	104	21.10%	21.10%
No	389	78.90%	78.90%
History of a similar previous episode:			
Yes	227	46%	46%
No	266	54%	54%
Priority given at the emergency department:			
1	49	9.90%	10.50%
2	105	21.30%	22.40%
3	121	24.50%	25.90%
4	180	36.50%	38.50%
5	13	2.60%	2.80%
Emergency Department visits in the past year:			
1-3 visits	344	69.80%	69.80%
4-7 visits	97	19.70%	19.70%
≥ 8 visits	52	10.50%	10.50%
Length of hospitalization:			
0 days (not admitted)	346	70.20%	70.20%
1-3 days	73	14.80%	14.80%
4-10 days	44	8.90%	8.90%
≥ 11 days	30	6.10%	6.10%
Known chronic disease:			
Hypertension	38	7.70%	7.70%
Diabetes	26	5.30%	5.30%
Both hypertension and diabetes	82	16.60%	16.60%
Epilepsy	90	18.30%	18.30%
Anemia	12	2.40%	2.40%
Chronic headache disease	5	1%	1%
Not known to have any chronic disease (medically healthy)	191	38.70%	38.70%
Diagnosis of COVID-19 at KAUH:			
Yes	25	5.10%	5.10%
No	468	94.90%	94.90%
Underwent brain imaging:			
Yes	182	36.90%	36.90%
No	311	63.10%	63.10%

**Table 2:** Comparison of patient characteristics between the pre-pandemic period and the pandemic period

(n)		PRE-PANDEMIC (percentage)		DURING PANDEMIC (percentage)	
Gender:					
Male		131 (47%)		104 (49%)	
Female		150 (53%)		108 (51%)	
Nationality:					
Saudi		181 (64%)		104 (49%)	
Non-Saudi		100 (36%)		108 (51%)	
Age group:					
0-14 years		34 (12%)		32 (15%)	
15-64 years		191 (68%)		140 (66%)	
≥ 65 years		56 (20%)		40 (19%)	
Known chronic disease:					
Hypertension		23 (10%)		15 (8%)	
Diabetes		18 (7%)		8 (4%)	
Both hypertension and diabetes		45 (18%)		37 (19%)	
Epilepsy		55 (22%)		35 (18%)	
Anemia		6 (2%)		6 (3%)	
Chronic headache disease		4 (1%)		1 (1%)	
Not known to have any chronic disease		102 (40%)		89 (47%)	
Reason for visit:					
Headache		134 (49%)		86 (42%)	
Seizures		70 (25%)		48 (23%)	
Weakness		45 (16%)		41 (20%)	
Dizziness		9 (3%)		12 (6%)	
Decreased LOC		13 (5%)		11 (5%)	
Other neurological complaints		5 (2%)		8 (4%)	
History of a similar previous episode:					
Yes		132(47%)		95 (45%)	
No		149 (53%)		117 (55%)	
Priority given at the emergency department:					
1		23 (9%)		26 (13%)	
2		61 (23%)		44 (22%)	
3		69 (26%)		52 (25%)	
4		107 (41%)		73 (36%)	
5		4 (2%)		9 (8%)	
Emergency Department visits in the past year:					
1-3 visits		200 (71%)		144 (68%)	
4-7 visits		55 (20%)		42 (20%)	
≥ 8 visits		26 (9%)		26 (12%)	
Length of hospitalization:					
0 days (not admitted)		197 (70%)		149 (70%)	
1-3 days		46 (16%)		27 (13%)	
4-10 days		20 (7.5%)		24 (11%)	
≥ 11 days		18 (6.5%)		12 (6%)	
Underwent brain imaging:					
Yes		95 (34%)		87 (41%)	
No		186 (66%)		125 (59%)	
Type of imaging performed:					
Brain CT scan		81 (85%)		84 (97%)	

**Figure 1:**
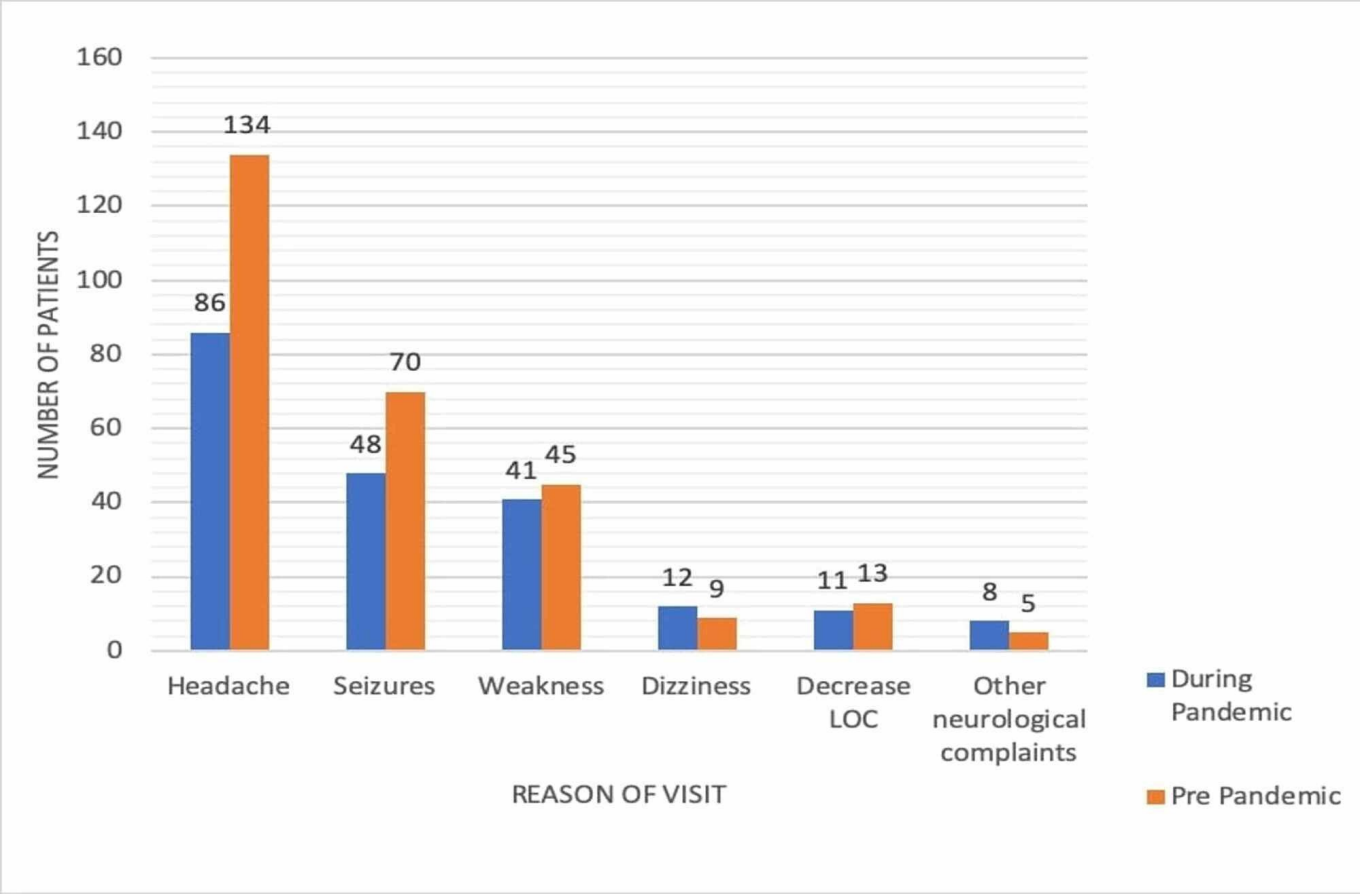
Neurological symptoms in different time periods

There was no significant relationship between the time period and the reason for the visit (p=0.305). The exact numbers presented in the two different time periods for each neurological symptom are shown in Figure 1. Brain computed tomography (CT) scans were the most ordered type of imaging (90.7%), which underwent an 11.3% increase during the pandemic time period (p=0.005). The number of visits for non-Saudi Arabian patients showed a 15.3% increase during the pandemic time period while there was a 15.3% decrease in the number of visits for Saudi Arabian patients.

There was no significant relationship between the time period and the priority given at the emergency department (p=0.193).

Our investigation provided a full description of different variables in two different time periods (pre-COVID-19 and during-COVID-19). It also showed significant relationships between the time period and type of brain imaging study and the nationality of the patient.

## Discussion

COVID-19 is a global pandemic affecting hospitals worldwide, the emergency department (ED) is one of if not the most heavily impacted hospital departments during this global pandemic, with that being said neurological symptoms are commonly presented in the emergency department, some of which can be life-disabling or even life-threatening. Our study applies a descriptive analysis of ED visits regarding common neurological conditions during two different time periods.

In our study, we found out that there was a 24.5% reduction in the number of visits for the common neurological conditions aforementioned in the during-the-pandemic time period in comparison to the pre-pandemic. While we have not found other studies assessing the effect of the time period (pre-COVID-19 versus during-COVID-19) on the number of visits regarding different common neurological conditions, we found other similar studies reporting a decline in the number of visits during the COVID-19 pandemic. For example, a study conducted in a trauma center in New Zealand, which looked at admission rates before and during government lockdown due to COVID-19 revealed a 48% reduction in all injury-related admissions [21], another study conducted in Canada showed a 20% decrease in the number of code strokes in the first few months of 2020 as compared to the equivalent period during 2019 [24]. Another study conducted in Italy reported an overall 73% decrease in the ophthalmological emergency department [26]. In addition, one study conducted in the United States of America, which compared the number of ED visits for acute and life-threatening conditions in the pre- and during-the-COVID-19 period noted a decrease of 23%, 20%, and 10% in ED visits regarding myocardial infarction, stroke, and hyperglycemic crises, respectively [27]. This may be due to different reasons, for instance, the government's issuing lockdown would cause people to stay at home unless their symptoms were an uttermost emergency, which varies depending on the person. Public anxiety regarding the pandemic may be another possible reason, as more people are practicing social distancing and avoiding crowded areas and places that are thought to carry a higher risk of COVID-19 exposure, instead, they try to manage non-urgent symptoms alone or by contacting medical personnel via telephone or mobile applications [26].

Our study also showed a significant increase in the rates of emergency brain CT scans in the during-COVID-19 time period compared to the (pre-COVID-19) time period. To our knowledge, no previous studies have assessed these two variables yet. In our opinion, this increase could be due to the fact that the ED physicians during the COVID-19 pandemic are both physically and mentally engaged in dealing with COVID-19-related concerns, therefore, when other non-COVID-19 related symptoms or conditions present to the ED; in this case, neurological, we believe they would be accordingly presumed as life-threatening until proven otherwise.

Our study further demonstrated a significant increase in the number of visits for non-Saudi Arabian patients in the during-pandemic time period. We think that this increase would be due to the fact that the Saudi Arabian government placed an order to treat all COVID-19 patients free of charge, regardless of their nationality, in both private and public hospitals. This may have caused Non-Saudi Arabian patients to have a lower threshold for visiting the emergency department at King Abdul-Aziz University Hospital (KAUH) for various conditions both COVID-19 and non-COVID-19-related.

Limitations and further suggestions

As this was a single-centered, retrospective record review of a terse period of time during the COVID-19 pandemic, only three months were included; therefore, it may not be generalized to other centers. Our choice to compare the COVID-19 months with the immediately preceding months together with choosing March 1 as our start date of the COVID-19 pandemic period were somewhat arbitrary. However, these choices were made based on a couple of factors, the first being that March witnessed an increasing amount of public health awareness campaigns regarding COVID-19 as compared to the preceding months, it was also on March 11 that the WHO declared the COVID-19 outbreak as a global pandemic. Another important factor is that the first confirmed positive COVID-19 case in Saudi Arabia was reported on March 8; furthermore, the Saudi Arabian government issued the first lockdown order on March 23. Because our study was a retrospective record review, we were limited by poor documentation, which also affected our ability to document the exact outcome of each visit for each patient.

We encourage future researchers to look into the confirmed COVID-19 cases that initially presented with neurological symptoms. They may also want to expand the time frame possibly comparing the total visits during 2019 with 2020.

## Conclusions

More and more studies are emerging regarding this global pandemic and its possible neurological sequelae. We do not have to ignore the community-related impact of this pandemic on ED visits for common neurological symptoms. In this study, we observed a decline in the number of emergency department visits for common neurological symptoms. Patients with some serious conditions are delaying or even withholding their appropriate needed care, which is, in fact, a very consequential matter and represents a serious public health concern. Therefore, it is essential to educate the population about the urgent neurological conditions that require prompt care and the seriousness of delaying the needed care. Furthermore, with the current healthcare focus on battling this global pandemic, it may be possible that some non-COVID-19-related patients may not be receiving the legitimate necessary care for their specific conditions. Therefore, campaigns promoting different ways to avoid these consequences are needed. In conclusion, although we have looked into some aspects of how the ED visits for common neurological conditions can be affected during the COVID-19 pandemic, there is still, in our opinion, a sea of future studies that can help make us understand and possibly avoid certain consequences, as the medical field has definitely suffered and will continue to suffer from the mass effect of this global pandemic, which has various aspects that research till date has barely scratched the surface of.
